# Postharvest UV-B Irradiation Stimulated Ginsenoside Rg_1_ Biosynthesis through Nitric Oxide (NO) and Jasmonic Acid (JA) in *Panax quinquefolius* Roots

**DOI:** 10.3390/molecules24081462

**Published:** 2019-04-13

**Authors:** Jie Zhou, Zhi-fang Ran, Xiao-tong Yang, Jia Li

**Affiliations:** 1 School of Biological Science and Technology, University of Jinan, Jinan 250022, China; 2 School of Pharmaceutical Sciences, Shandong University of Traditional Chinese Medicine, Jinan 250355, China; ranzhifang429220@126.com (Z.-f.R.); 13476223357@163.com (X.-t.Y.)

**Keywords:** postharvest UV-B radiation, *Panax quinquefolius*, Rg_1_, nitric oxide, jasmonic acid

## Abstract

The study highlights the influence and signal transduction mechanism of postharvest UV-B on the production of Rg_1_ in *Panax quinquefolius* roots during the drying process. The results showed that postharvest UV-B irradiation induced generation of nitric oxide (NO), jasmonic acid (JA), and ginsenoside Rg_1_ of *P. quinquefolius* roots. The UV-B-induced increase of Rg_1_ was suppressed by NO-specific scavenger (cPTIO) and NOS inhibitors (PBITU), JA synthesis inhibitor (SHAM), and JA synthesis inhibitor (PrGall), indicating that NO and JA played essential parts in UV-B-induced Rg_1_. External NO inhibitors treatment inhibited UV-B-induced accumulation of NO and JA, which suggested that NO was located upstream of the JA signal pathway. NO-caused Rg_1_ was inhibited by SHAM and PrGall, implying JA participated in transmitting signal NO to Rg_1_ accumulation. In other words, NO mediated the postharvest UV-B-induced Rg_1_ accumulation by the JA-dependent pathway in *P. quinquefolius* roots during the drying process, which helps us understand the underlying mechanisms involved in UV-B-induced Rg_1_ production and provides information helpful for *P. quinquefolius* production.

## 1. Introduction

American ginseng (*Panax quinquefolius* L.), which is native to North America, has been used for medicinal purposes for centuries. In China, its dried roots have been used extensively for the treatment of cardiovascular, endocrine, immune, and central nervous system diseases in traditional Chinese medicines (TCM) for about 300 years [1,2]. Ginsenosides (including Rg_1_), as secondary metabolites in *P. quinquefolius* roots, are the major bioactive components and important indexes for measuring the quality of American ginseng [3,4]. In recent years, American ginseng has been widely used in medicine, food, and cosmetics, which has increased the demand for *P. quinquefolius*. Furthermore, the current supply to the market almost depends on the field cultivation, thus great efforts have been made to improve the content of bioactive components in *P. quinquefolius* roots.

It is well known that the pharmacological value of TCM depends highly on the postharvest processing steps, such as temperature, heating light, or UV-B irradiation [5,6]. The potential use of UV-B irradiation as an effective measure to improve the bioactive materials in vegetables and fruits has been well investigated [7,8,9,10]. The accumulation of phenol and expression of related genes in peaches have been improved by postharvest UV-B irradiation [11]. The biosynthesis of polyphenols in white cabbage leaves has been stimulated by UV-B irradiation, which has been regarded as a supplemental measure to improve the nutritional quality of vegetables [5]. Until now, the effects and potential mechanisms of postharvest UV-B irradiation on the accumulation of ginsenosides in *P. quinquefolius* roots have not been reported.

The activation of endogenous signal transduction has been well investigated and was found to be an important mechanism involved in the biosynthesis of secondary metabolites. Numerous physiological responses, comprising nitric oxide (NO), jasmonic acid (JA), and so on, have been associated with biosynthesis of secondary metabolites in medicinal plants. NO has been reported to participate in UV-B-induced flavonoids accumulation in *Betula pendula* and isoflavone biosynthesis in soybean sprouts [12,13]. JA has been also considered as an essential signal molecule in UV-B-induced biosynthesis of defensive compounds in soybeans [14]. However, so far, no information is available in the literature about the signaling mechanism of postharvest UV-B-induced ginsenosides in *P. quinquefolius* roots. Therefore, the present study deals with the investigation on the influence of postharvest UV-B on the biosynthesis of Rg_1_ and the possible parts of NO and JA in UV-B-induced Rg_1_ production in *P. quinquefolius* roots.

## 2. Results and Discussion

### 2.1. Effects of UV-B Irradiation on Accumulation of Rg_1_

**Figure 1:**
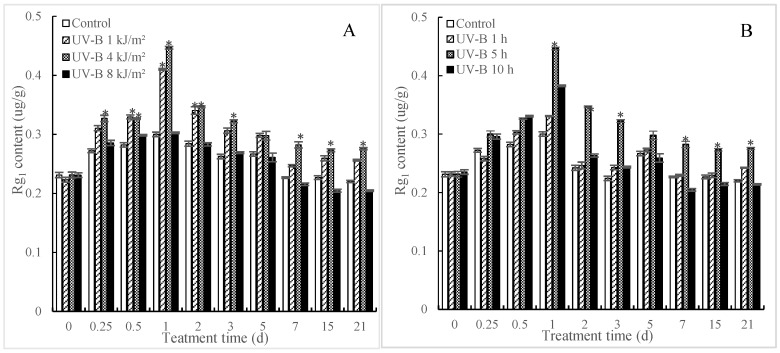
Effects of UV-B irradiation on Rg_1_ content. The *P. quinquefolius* roots that were irradiated with different doses of UV-B (**A**) or irradiated with 4 kJ/m^2^ UV-B for different times (**B**) were taken for measuring the accumulation of Rg_1_ in *P. quinquefolius* roots during a 21-day postharvest drying process. Data are means of three replicates ± SD. Asterisks show significant differences (*p* < 0.05, *t*-test) for the samples between UV-B treatment and control sampled at the same time point according to one-way ANOVA followed by Student–Newman–Keuls tests (*p* < 0.05).

In order to enhance the production of Rg_1_, we investigated the effect of UV-B irradiation on Rg_1_ accumulation in *P. quinquefolius* roots during the postharvest drying process. As shown in Figure 1A, the content of Rg_1_ in *P. quinquefolius* roots showed an increase in the earlier phase (within 24 h after UV-B treatment), while it showed a downtrend in the latter stage (after 24 h by UV-B treatment) in both the control and treated groups. Treatments with a lower dose of UV-B resulted in significant increases in Rg_1_ accumulation, which were evaluated as being 36.73% (1 kJ/m^2^ UV-B) and 48.89% (4 kJ/m^2^ UV-B) higher (*p* < 0.05) than those in the control on 1 d after UV-B treatment. Treatment with a higher dose of UV-B (8 kJ/m^2^) slightly inhibited Rg_1_ accumulation, suggesting that the effects of UV-B on the accumulation of Rg_1_ in *P. quinquefolius* roots was dose-dependent (Figure 1B). The influence of duration of UV-B irradiation on the accumulation of Rg_1_ was also investigated. Figure 1B displayed that roots treated by 4 kJ/m^2^ UV-B for 5 h had the highest (*p* < 0.05) content of Rg_1_ compared with other exposure times, so this dose was chosen for the following experiments in this study. 

The postharvest drying process is actually a physiological process against “drought stress” for freshly collected roots, so these roots would activate the anti-drought pathway and improve the content of related secondary metabolites during the postharvest drying process. In this study, the value of Rg_1_ in *P. quinquefolius* roots showed an inverse V-shape, that is, upgraded at first and then degraded during the postharvest drying process. It remains to be further confirmed whether Rg_1_ plays a role in the anti-drought physiological process of *P. quinquefolius*. In addition, postharvest UV-B treatment is often necessary to improve the accumulation of secondary metabolite in plants. Wei et al. [15] reported that UV-B had a long-lasting influence on the levels of secondary metabolites in pigeonpea leaves. Schreiner et al. [16] found that the concentration of phenolic was improved to be six-fold that in the control by treatment of UV-B. Our results indicated that a suitable dose of UV-B irradiation (4 kJ/m^2^ UV-B for 5 h) might be a potential tool to improve the accumulation of Rg_1_ in the *P. quinquefolius* roots in the postharvest drying progress. 

### 2.2. Effects of UV-B Irradiation on NO Generation

**Figure 2:**
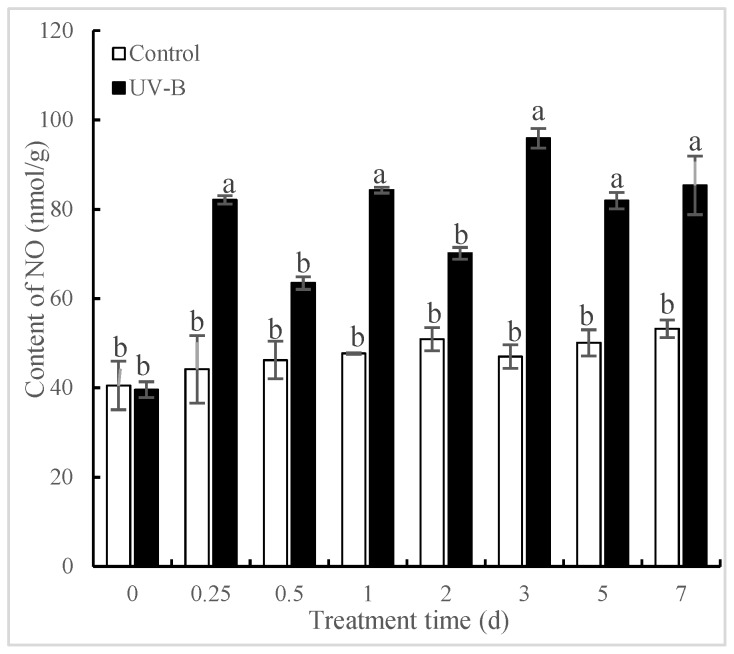
Effects of UV-B irradiation on NO content of *P. quinquefolius* roots. The *P. quinquefolius* roots that were irradiated with 4 kJ/m^2^ UV-B for 5 h were taken for measuring nitric oxide (NO) and jasmonic acid (JA) contents during a 21-day postharvest drying process. Data are means ± SE of three replicates. Different letters indicate significantly different values according to one-way ANOVA followed by Student–Newman–Keuls tests (*p* < 0.05).

Numerous studies have shown that short-term UV-B irradiation is such a “stress” that could affect the physiological process of plants [17,18]. An array of defense responses in plants such as accumulation of signal molecules could be caused in response to “stress”. NO as a key signal molecule regulates physiological metabolism and defense responses against stresses in plants [19]. The time courses of NO levels in *P. quinquefolius* roots after exposure to UV-B irradiation are exhibited in Figure 2. The results indicated that the content of NO in the *P. quinquefolius* roots increased over time, reaching 85.95%, significantly more than that in the control at 6 h (*p* < 0.05), 76.69% more at 24 h and 104.03% more at 3 d (*p* < 0.05) after UV-B irradiation. The content of NO in untreated groups did not show dramatic changes during the postharvest drying process, suggesting that the generation of NO in *P. quinquefolius* roots was not attributable to development-dependent changes. Yu et al. [20] found that NO was involved in the fungal elicitors-induced increase of ginsenoside in *P. quinquefolius* roots. It is revealed that NO participated in elicitor oligogalacturonic acid (OGA)-induced saponin accumulation in *Panax ginseng* [19]. In our study, it is hypothesized that NO may serve as a signal molecule in the UV-B-induced increase of Rg_1_ in *P. quinquefolius* roots.

### 2.3. Effects of UV-B Irradiation on JA Generation

**Figure 3:**
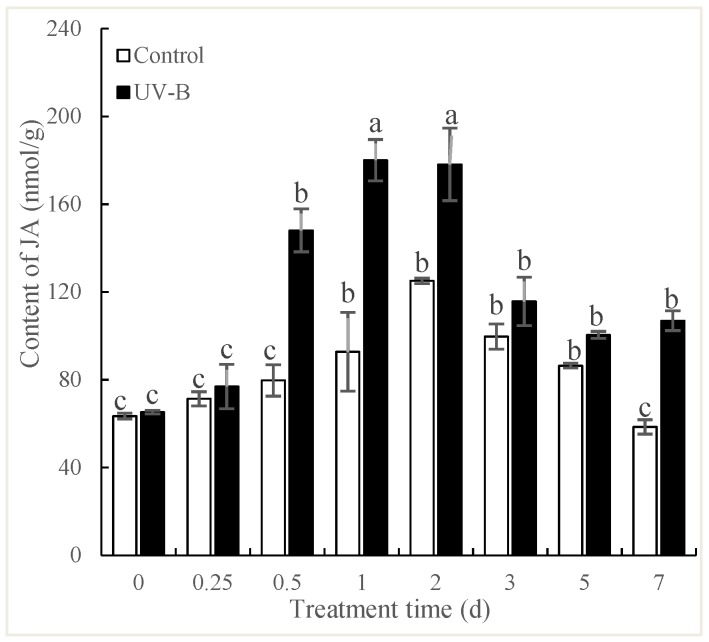
Effects of UV-B irradiation on JA content of *P. quinquefolius* roots. The *P. quinquefolius* roots that were irradiated with 4 kJ/m^2^ UV-B for 5 h were taken for measuring NO and JA contents during a 21-day postharvest drying process. Data are means ± SE of three replicates. Different letters indicate significantly different values according to one-way ANOVA followed by Student–Newman–Keuls tests (*p* < 0.05).

UV-B irradiation could induce generation of signal molecules such as NO and JA, which are integral parts of signal transduction pathway involved in plant defense response [21,22]. As shown in Figure 3, the content of JA in *P. quinquefolius* roots significantly increased over time, reaching a peak at 1.94-fold (*p* < 0.05) of that in the control at 24 h after UV-B treatment, and then decreased gradually, but remained significantly higher (*p* < 0.05) than that of the control. It has been well investigated that JA production is one of the common responses to UV-B in plants [23]. Menke et al. [24] demonstrated that JA participated in the fungal elicitor-induced expression of key genes in terpenoid indole alkaloid biosynthesis in *Catharanthus roseus* cells. In our study, the UV-B induced increase of JA occurred later than NO in *P. quinquefolius* roots.

### 2.4. Dependence of UV-B-Induced Increase of Rg_1_ on NO and JA

**Figure 4:**
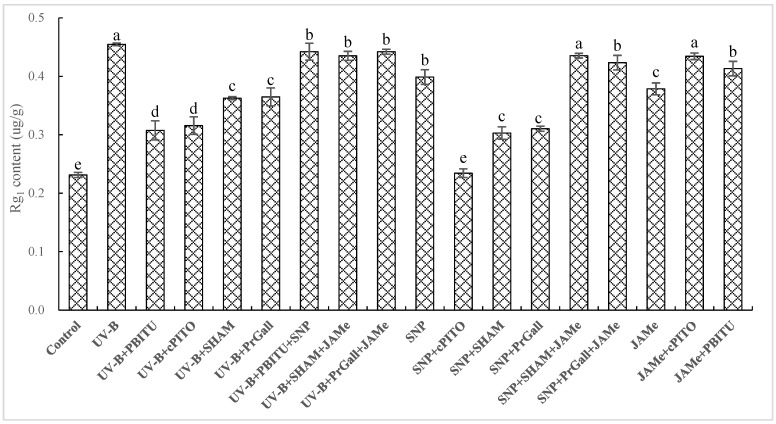
Effects of inhibitors on the UV-B-induced Rg_1_ production of *P. quinquefolius* roots. The roots treated with UV-B irradiation and inhibitors were harvested at 24 h after UV-B irradiation, and Rg_1_ production was then determined. Inhibitors were pretreated 1 h before UV-B irradiation. The control received the vehicle solvent only. Data are means of three replicates ± SD. Different letters indicate significantly different values according to one-way ANOVA followed by Student–Newman–Keuls tests (*p* < 0.05).

The production of NO and JA as earlier biochemical reaction responding to UV-B was shown in our experiments. To investigate whether NO and JA participated in UV-B-induced accumulation of Rg_1_ in *P. quinquefolius* roots, the effects of scavengers and inhibitors of JA and NO on UV-B-induced Rg_1_ were determined (Figure 4). The UV-B-induced increase of Rg_1_ was significantly (*p* < 0.05) inhibited by treatments of NO-specific scavenger cPTIO and PBITU. In our results, UV-B-induced increase of Rg_1_ depended on NO generation; in other words, NO was considered as an upstream signal molecule in the pathway of the UV-B-induced increase of Rg_1_. Treatments with JA synthesis inhibitors SHAM and PrGall resulted in a decline in Rg_1_ content. This exhibited that JA played an important role in the UV-B-induced increase of Rg_1_ in *P. quinquefolius* roots. These results were further confirmed by the finding that the suppression of inhibitors of JA and NO on UV-B-induced Rg_1_ was turned back by external JA donor jasmonic acid methyl ester (JAMe) and NO donor sodium nitroprusside (SNP).

### 2.5. Dependence of UV-B-Induced JA Biosynthesis on NO 

**Figure 5:**
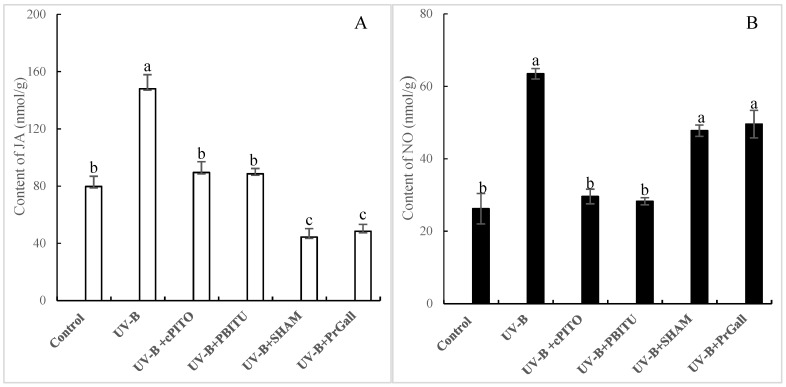
Effects of inhibitors on UV-B-induced jasmonic acid (JA) generation (**A**) and nitric oxide (NO) accumulation (**B**) of *P.* *quinquefolius* roots. The roots treated with UV-B irradiation and various inhibitors were harvested at 12 h after UV-B irradiation, and NO and JA contents were determined. Inhibitors were pretreated 1 h before UV-B irradiation. The control received the vehicle solvent only. Data are means of three replicates ± SD. Different letters indicate significantly different values according to one-way ANOVA followed by Student–Newman–Keuls tests (*p* < 0.05).

The fact that JA and NO was involved in the UV-B-induced Rg_1_ biosynthesis in roots of *P. quinquefolius* was shown in our experiments. To evaluate the upstream and downstream relationship between NO and JA, the influence of PBITU and cPITO on UV-B-induced JA and SHAM and PrGall on UV-B-induced NO was investigated. These results displayed that UV-B-induced JA burst was significantly depressed by cPITO and PBITU (*p* < 0.05, Figure 5A); however, UV-B-induced NO was not observed to be severely affected by SHAM and PrGall (Figure 5B). These data indicated that NO played an important role in UV-B-induced JA biosynthesis.

### 2.6. Dependence of NO-Induced Rg_1_ Production on JA

As displayed in Figure 4, the content of Rg_1_ was significantly improved (*p* < 0.05) by NO donor SNP, exceeding as much as 87.70% of that of the UV-B response. The SNP-induced increase of Rg_1_ was significantly blocked by SHAM and PrGall. NO scavengers PBITU and cPITO did not inhibit the JA-induced increase of Rg_1_. It wass revealed that NO-triggered Rg_1_ production depended on JA. Moreover, the suppression of SHAM and PrGall on SNP-induced Rg_1_ is relieved by external treatment of JAMe.

## 3. Materials and Methods

### 3.1. Plant Materials and Experimental Design

The roots of *P. quinquefolius* aged four years old were collected from a *P. quinquefolius* planting base from Weihai, Shandong Province, PR China, at the time of natural dispersal on November 10, 2017. The healthy roots with uniform size were immediately sent to the laboratory after harvest within 6 h. The roots were treated with UV-B irradiation (TL 20W/01 RS, Philips, Amsterdam, Netherlands) with doses of 1, 4, and 8 kJ/m^2^, and the designated irradiation doses were realized by regulating the distance from roots to lamp tubes and altering the exposure time. Each root was overturned 180° to obtain uniform irradiation on their back during the exposure process [25]. Following treatments, the roots in both the control and the treated group were respectively divided into two groups; Following treatments, the roots in both the control and the treated group were sampled at the adaptation time point and respectively divided into two groups. One group was placed at room temperature and air-dried (25 °C, 50% relative humidity) for determination of Rg_1_. The aliquot of the material was frozen in liquid nitrogen and stored at −80 °C for further analysis. The chemical reagent used in the experiment was mainly obtained from Sigma-Aldrich, including NO donor sodium nitroprusside (SNP), scavenger of NO-specific2-(4-carboxyphenyl)-4,4,5,5-tetramethylimidazoline-1-oxyl-3-oxide (cPTIO), inhibitors of nitric oxide synthase (NOS), S,S′-1,3-phenylene-bis (1,2-ethanediyl)-bis-isothiourea (PBITU), NG-nitrol-Arg methyl ester (l-NAME), inhibitors of nitrite reductase (NR), tungstate (TUN), and glutamine (Gln). Jasmonic acid methyl ester (JAMe), JA synthesis inhibitor salicylhydroxamic acid (SHAM), and JA synthesis inhibitor n-propylgallate (PrGall) [26,27]. The exogenous signaling molecules and inhibitors were dissolved in water or 0.2% dimethyl sulfoxide solution and were sprayed evenly on roots until excess drops began to fall 1 d before application of UV-B-or signaling molecules treatments, unless stated otherwise. Each treatment consisted of 10 replicates, and all treatments were repeated three times.

### 3.2. Measurement of JA

The content of JA was measured and calculated according to the method of Pan et al. [28]. The powder (500 mg) of *P. quinquefolius* roots, which was obtained in a mortar and pestle with liquid nitrogen, was put into a centrifuge tube with 2 mL of extraction solvent (n-propanol/water/concentrated hydrochloric acid: 200/100/0.2, v) and placed on a shaker with a speed of 100 rpm for 30 min at 4 °C. Then, 2 mL dichloromethane was added the sample, after which it was shaken gently and centrifuged for 15 min (4 °C, 3500 rpm). The residue was added to 0.5 mL dichloromethane and vortex for 30 s, and then two supernatants were combined and concentrated with a nitrogen evaporator. The samples were dissolved in 0.5 mL methanol and injected into 50 μL sample solution for high-performance liquid chromatography (HPLC) analysis. 

### 3.3. Measurement of NO

The extracts of NO were prepared by homogenizing 0.5 g of *P. quinquefolius* roots in a mortar on ice with 1.0 mL phosphate buffer saline. The content of NO was determined by the method described in the instruction manual of the kit (Shanghai Enzyme Biotechnology Co., Ltd., Shanghai, China). 

### 3.4. HPLC Analysis of Rg_1_

The content of Rg_1_ was measured using high-performance liquid chromatography (HPLC) according to the method described by Li et al. [29]. The dried powder roots of *P. quinquefolius* (500 mg) were extracted with 10 mL methanol. The extracts were treated with ultrasound for 40 min and then placed stably for 10 min. A 0.45 um microporous membrane was used to filter the supernatant, and 10 µL was injected for HPLC analysis. The HPLC analysis was carried on a Kromasil C18 (4.6 × 250 mm, 5 µm) column, the mobile phase composed of acetonitrile/water/0.1% phosphoric acid solution (20:40:40) at a speed of 1.0 mL/min. The wavelength was monitored at 203 nm and the column component was kept at 40 °C. The content of Rg_1_ was obtained according to the regression equation of standard curve.

### 3.5. Statistical Analysis

The mean and standard deviation were calculated for each biochemical measurement. One-way analysis of variance (ANOVA) was carried out using SPSS (version 18.0, SPSS, Inc., Chicago, IL, USA) statistical software, and statistical differences (*p* < 0.05) between means of pairs were resolved using confidence intervals using Student–Newman–Keuls tests. 

## 4. Conclusions

In summary, the data obtained from this work demonstrated that postharvest UV-B with a dose of 4 kJ/m^2^ for 5 h could improve the accumulation of Rg_1_. JA and NO played signal roles in UV-B induced accumulation of Rg_1_. NO regulated the UV-B-induced Rg_1_ through a JA-dependent signaling pathway. Together, the results suggested postharvest UV-B radiation might be used as a new practical approach to improve Rg_1_ accumulation by modulating NO and JA in *P. quinquefolius* roots.
